# Multicentre observational study of quality of life after surgical palliation of malignant gastric outlet obstruction for gastric cancer

**DOI:** 10.1002/bjs5.26

**Published:** 2018-03-15

**Authors:** K. Fujitani, M. Ando, K. Sakamaki, M. Terashima, R. Kawabata, Y. Ito, T. Yoshikawa, M. Kondo, Y. Kodera, K. Yoshida

**Affiliations:** ^1^ Department of Surgery Osaka Prefectural General Medical Centre Osaka Japan; ^2^ Centre for Advanced Medicine and Clinical Research Nagoya University Hospital Nagoya Japan; ^3^ Department of Gastroenterological Surgery Aichi Cancer Centre Nagoya Japan; ^4^ Department of Gastroenterological Surgery Nagoya University Graduate School of Medicine Nagoya Japan; ^5^ Department of Biostatistics Yokohama City University Graduate School of Medicine Yokohama Japan; ^6^ Department of Gastrointestinal Surgery Kanagawa Cancer Centre Yokohama Japan; ^7^ Division of Gastric Surgery Shizuoka Cancer Centre Nagaizumi Japan; ^8^ Department of Surgery Osaka Rosai Hospital Sakai Japan; ^9^ Department of Surgery Kobe City Medical Centre General Hospital Kobe Japan; ^10^ Department of Surgical Oncology Gifu University Graduate School of Medicine Gifu Japan

## Abstract

**Background:**

Quality of life (QoL) is a key component in decision‐making for surgical palliation, but QoL data in association with surgical palliation in advanced gastric cancer are scarce. The aim of this multicentre observational study was to examine the impact of surgical palliation on QoL in advanced gastric cancer.

**Methods:**

The study included patients with gastric outlet obstruction caused by incurable advanced primary gastric cancer who had no oral intake or liquid intake only. Patients underwent palliative distal/total gastrectomy or bypass surgery at the physician's discretion. The primary endpoint was change in QoL assessed at baseline, 14 days, 1 month and 3 months following surgical palliation by means of the EuroQoL Five Dimensions (EQ‐5D™) questionnaire and the European Organisation for Research and Treatment of Cancer Quality of Life Questionnaire gastric cancer module (QLQ‐STO22). Secondary endpoints were postoperative improvement in oral intake and surgical complications.

**Results:**

Some 104 patients (23 distal gastrectomy, 9 total gastrectomy, 70 gastrojejunostomy, 2 exploratory laparotomy) were enrolled from 35 institutions. The mean EQ‐5D™ utility index scores remained consistent, with a baseline score of 0·74 and the change from baseline within ± 0·05. Gastric‐specific symptoms showed statistically significant improvement from baseline. The majority of patients were able to eat solid food 2 weeks after surgery and tolerated it thereafter. The rate of overall morbidity of grade III or more according to the Clavien–Dindo classification was 9·6 per cent (10 patients) and the 30‐day postoperative mortality rate was 1·9 per cent (2 patients).

**Conclusion:**

In patients with gastric outlet obstruction caused by advanced gastric cancer, surgical palliation maintained QoL while improving solid food intake, with acceptable morbidity for at least the first 3 months after surgery. Registration number 000023494 (UMIN Clinical Trials Registry).

## Introduction

Gastric cancer is the third leading cause of cancer‐related death and the fifth most common cancer diagnosed worldwide.^1^ The prognosis of patients with incurable advanced gastric cancer is dismal and most die within 1 year^2^. In addition, patients with incurable advanced gastric cancer often present with various clinical symptoms such as abdominal pain/distension, anorexia, weight loss and nausea/vomiting^3^. They have poor oral intake, often related to mechanical gastric outlet obstruction. Although palliative operative procedures have been used for many years, the impact of surgical intervention on health‐related quality of life (QoL) in relieving symptoms, maintaining independence and function, allowing home care, and minimizing the burden for carers^4^ is still poorly understood. Decision‐making for surgical care is difficult. Age, performance status (PS), nutritional status, co‐morbidities, previous and future anticancer treatments, and postoperative morbidity and mortality all affect the outcome of surgical palliation. Ideally, surgical palliation should achieve durable symptom resolution without detriment to QoL or impairment of survival.

QoL assessment is now recognized as a key component in clinical evaluation^5^, particularly in patients with incurable advanced cancer in whom the scope for improving survival is limited. Although some data exist on QoL associated with palliative chemotherapy for advanced gastric cancer^6^, ^7^, ^8^, QoL data associated with surgical palliation are sparse^9^ ^10^. The aim of this study was to examine the impact of surgical palliation on postoperative QoL in patients with malignant gastric outlet obstruction caused by incurable advanced gastric cancer.

## Methods

This was a multicentre observational study conducted at 35 cancer centres, medical centres, university hospitals and general hospitals in Japan. The study protocol was approved by the institutional review board of each participating hospital before initiation of the study. The study was conducted in accordance with the international ethical recommendations stated in the Declaration of Helsinki, and the Japanese Ethical Guidelines for Clinical Research.

The primary endpoint was change in QoL assessed at 14 days, 1 month and 3 months following surgical palliation. Secondary endpoints were postoperative improvement in oral intake and surgical complications.

### Eligibility criteria

Eligibility criteria included: histologically proven primary gastric adenocarcinoma presenting with gastric outlet obstruction diagnosed clinically, endoscopically or radiographically; presence of non‐curable factors confirmed by both enhanced abdominal CT and exploratory laparoscopy or laparotomy; extremely poor or no oral intake requiring parenteral nutrition; age 20 years or more; surgically fit without severe cardiac or pulmonary dysfunction, or massive ascites, pleural effusion or oedema judged by the physician performing the surgical procedure; Eastern Cooperative Oncology Group (ECOG) PS score 0–2; adequate organ function within 14 days before enrolment, defined as leucocyte count at least 3·0 × 10^9^/l, haemoglobin level at least 80 g/l with or without transfusion, platelet count 100 × 10^9^/l or more, total bilirubin level 51·3 μmol/l or below and serum creatinine concentration no more than 176·8 μmol/l; and written informed consent.

Patients were excluded if they met any of the following criteria: active bleeding or perforation of the gastric tumour necessitating urgent surgery; active symptomatic coexisting cancer (synchronous coexisting cancer or recurrence of metachronous cancer); clinically suspected brain metastasis or carcinomatous meningitis; presence of disseminated intravascular coagulation; and severe psychiatric disorder.

### Surgical procedures

Within 14 days of enrolment, patients underwent surgical palliation by either distal/total gastrectomy or gastrojejunostomy. Further points of obstruction in the small bowel or colon leading to concurrent small bowel/colonic bypass, small bowel/colonic resection, ileostomy/colostomy and catheter ileostomy were all allowed, but placement of an endoscopic stent was not. The choice of treatment was left to the discretion of the physician performing the surgical procedure. A Devine bypass procedure with transection of the stomach and anastomosis between the jejunal loop and the proximal stump of the divided stomach^11^, or a modified procedure with gastric partitioning, was also allowed. Patients who underwent diagnostic laparotomy alone on the basis of intraoperative findings were included.

### Quality‐of‐life assessment and tolerance of oral intake

At the time of enrolment, patients completed baseline QoL surveys. Follow‐up QoL surveys were conducted in all surviving patients at 14 days, 1 month and 3 months after surgical palliation. Questionnaires were administered by research assistants by means of personal interviews with inpatients, and sent by post to outpatients with stamped return envelopes addressed to the study coordinator.

QoL was assessed using the EuroQoL Five Dimensions (EQ‐5D™; EuroQol Group, Rotterdam, The Netherlands) questionnaire^12^, ^13^, ^14^ and the European Organisation for Research and Treatment of Cancer (EORTC) Quality of Life Questionnaire gastric cancer module (QLQ‐STO22)^15^ ^16^. The EQ‐5D™ is a non‐specific self‐classifier with five questions (concerning mobility, self‐care, daily activities, pain/discomfort and mood); index scores ranging from −0·111 to 1·000 were calculated based on UK preference weights^13^, with high scores representing good health status. The QLQ‐STO22 is intended for patients at all disease stages undergoing surgical resection, palliative surgical intervention, endoscopic palliation or palliative chemotherapy. It consists of 22 validated questions that evaluate five multi‐item symptom scales (dysphagia, eating restrictions, pain, reflux and anxiety), and four single‐item symptoms scales (dry mouth, body image, hair loss and loss of taste). Scoring was performed according to the EORTC QLQ‐C30 Scoring Manual^17^; scores ranging from 0 to 100 were calculated when at least half of the items were completed by the patient. A lower score indicates better QoL, with a score of 0 representing no symptoms.

Patients were also asked to describe ability to tolerate oral intake at baseline, 14 days, 1 month and 3 months after surgical palliation, based on the Gastric Outlet Obstruction Scoring System (GOOSS): 0, no oral intake; 1, liquids only; 2, soft solids; and 3, low‐residue or full diet^18^.

### Evaluation of operative morbidity and mortality

Surgical and non‐surgical complications were diagnosed clinically, radiographically or endoscopically, and evaluated according to the Clavien–Dindo classification^19^ ^20^. Reoperation details and duration of hospital stay after palliative intervention until hospital discharge were recorded. Postoperative death from any cause within 30 days of surgery and hospital death during the same hospital stay were also recorded.

### Statistical analysis

The planned sample size was 100 to detect a measured QoL difference of 0·3 s.d. after palliation compared with before surgery, with a two‐sided α of 5 per cent and 80 per cent statistical power.

For EQ‐5D™ index scores and each subscale of the QLQ‐STO22, changes from baseline at each postoperative time point, both for the whole cohort and subdivided by type of surgery (gastrojejunostomy or gastrectomy), were compared using the Wilcoxon signed‐rank test for matched pairs.

To make the statistically significant results more meaningful to clinicians and patients, QoL scores were converted to reflect the proportion of patients with a clinically significant change in each instrument score. EQ‐5D™ scores characterized each assessment after baseline as improved or deteriorated if the score changed by at least 0·05 points, and stable if it changed by less than 0·05 points^21^, ^22^, ^23^.

All statistical analyses were undertaken using SAS® statistical software version 9.4 (SAS Institute, Cary, North Carolina, USA), and *P* < 0·050 was considered statistically significant.

## Results

A total of 115 patients consented to be included in the study. Of these, 11 were ineligible leaving 104 patients in the analysis. *Table* 1 shows the characteristics of the study participants. Seventy‐one patients were men, and the median age was 68 (i.q.r. 63–75) years. Fifty‐nine patients had a serum C‐reactive protein level within the normal range (0–3·0 mg/l). Only 20 and 47 patients had serum albumin (40–51 g/l) and haemoglobin (115–168 g/l) levels in the normal range respectively. Forty‐one patients presented with a low BMI (below 18·5 kg/m^2^). The number of patients with non‐curable factors, such as T4b tumour (tumour infiltrating adjacent organs), peritoneal metastasis (P1), positive peritoneal cytology (CY1), hepatic metastasis (H1) and distant metastasis (M1), are shown in *Table* 1. Seventy‐five patients had two or more factors present.

**Table 1:** Patient characteristics

	No. of patients*
ECOG performance status	
0–1	88
2	16
BMI (kg/m^2^)†	19·7 (17·8–22·3)
C‐reactive protein (mg/l)†	2·2 (0·9–9·5)
Albumin (g/l)†	33 (29–39)
Haemoglobin (g/l)†	112 (101–125)
Non‐curable factor	
Tumour infiltrating to adjacent organs (T4b)	60
Peritoneal metastasis (P1)	55
Positive peritoneal cytology (CY1)	63
Hepatic metastasis (H1)	19
Distant metastasis (M1)	28
Surgical procedure	
Gastrojejunostomy	70
Open	58
Laparoscopic	12
Distal gastrectomy	23
Total gastrectomy	9
Exploratory laparotomy	2

* Unless indicated otherwise;

† values are median (i.q.r.). ECOG, Eastern Cooperative Oncology Group.

Types of surgery are shown in *Table* 1. Concurrent small bowel/colonic bypass was undertaken in nine patients, small bowel/colonic resection in two and ileostomy/colostomy in five patients. All gastrectomies were carried out by open surgery. Median duration of surgery and operative blood loss were 141 (i.q.r. 110–193) min and 30 (8–158) ml respectively.

### Quality‐of‐life changes

Baseline QoL questionnaires were completed by all patients before any intervention. At 14 days, 98 patients (94·2 per cent) completed the QoL questionnaires, and there were six non‐responders. At 1 month, 100 (96·2 per cent) completed the questionnaires; two did not respond and two had died. At 3 months, 82 (78·8 per cent) completed the QoL questionnaires; there were six non‐responders and 16 patients had died. Seventy‐seven patients (74·0 per cent) completed the questionnaires at all follow‐up points. The rate of QoL questionnaire completion was similar in patients undergoing gastrectomy and those having gastrojejunostomy.

**Figure 1:**
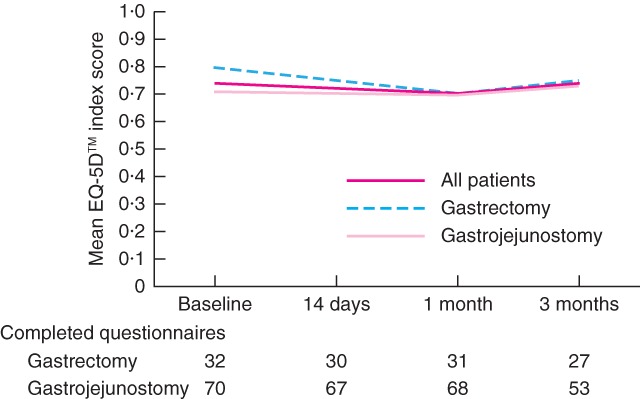
Changes in EuroQoL Five Dimensions (EQ‐5D™) scores after surgery. The index score ranges from −0·111 to 1·000, with high scores representing good health status

The primary endpoint, a measured QoL difference of 0·3 s.d. after surgical palliation, was not met. The mean(s.d.) baseline EQ‐5D™ score of all patients was 0·74(0·21) (*Fig*. 1). During follow‐up, mean scores remained consistent with baseline scores. The change from baseline score was within ± 0·05 for all patients and comparable between the two surgical procedures throughout the study.

**Figure 2:**
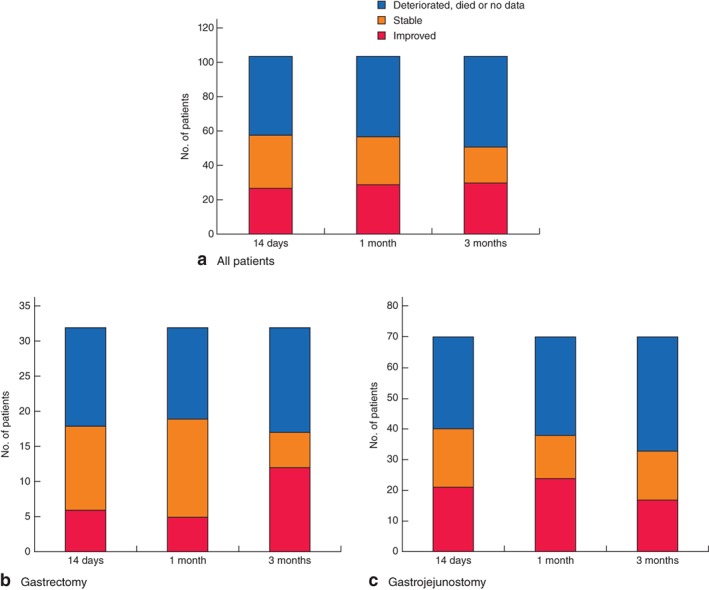
Number of patients with improved or stable EuroQoL Five Dimensions (EQ‐5D™) scores after surgery, compared with number whose scores deteriorated, or who died or had no data available: **a** all 104 patients, **b** 32 patients who underwent gastrectomy and **c** 70 patients who underwent gastrojejunostomy. The EQ‐5D™ scores at each postoperative assessment were considered to have improved or deteriorated if the score changed by at least 0·05 points, and stable if it changed by less than 0·05 points

As patients who returned no data might be those who were most ill, at each assessment point they were included in a combined group, allowing a comparison to be made between patients with improved or stable EQ‐5D scores™ versus those with deteriorated scores or who had died or had no data available at each time point (Fig. 2 a). Some 46 of 104 patients (44·2 per cent) could have experienced deterioration in QoL shortly after surgical palliation at 14 days after surgery, and the percentages were 45·2 per cent at 1 month and 51·0 per cent at 3 months. A greater percentage of patients with gastrectomy were classified as improved compared with those with gastrojejunostomy for EQ‐5D™ scores at 3 months (Fig. 2 b,c).

**Figure 3:**
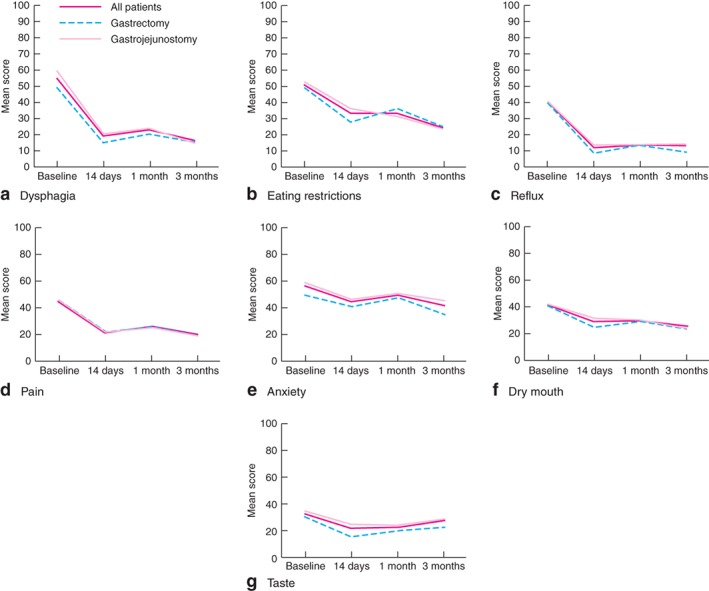
Changes in European Organisation for Research and Treatment of Cancer Quality of Life Questionnaire gastric cancer module (QLQ‐STO22) scores: **a** dysphagia, **b** eating restrictions, **c** reflux, **d** pain, **e** anxiety, **f** dry mouth and **g** taste. Scores range from 0 to 100, with low scores representing less symptom burden

Among gastric‐specific symptoms assessed by QLQ‐STO22, dysphagia, reflux, eating restrictions, pain, anxiety and dry mouth showed a statistically significant improvement from baseline at all assessment points in patients who underwent either gastrectomy or gastrojejunostomy (*P* < 0·001) (*Fig*. 3). In contrast, taste at 3 months was not altered by either intervention (*P* = 0·635).

### Improvement in oral intake

**Figure 4:**
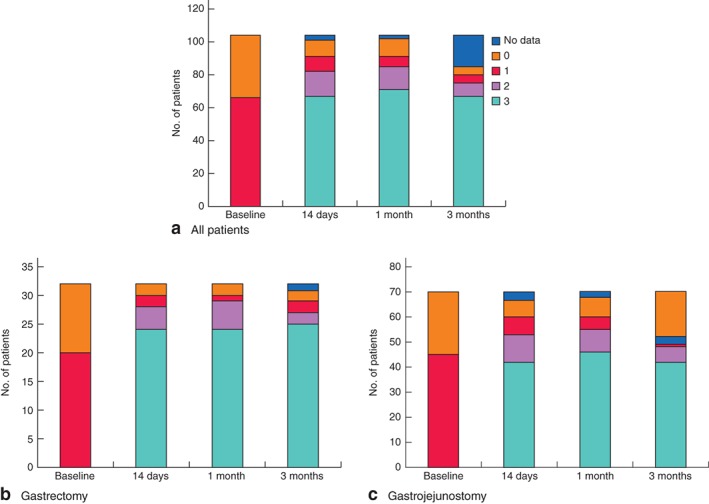
Changes in oral intake after surgery based on the Gastric Outlet Obstruction Scoring System (0, no oral intake; 1, liquids only; 2, soft solids; 3, low‐residue or full diet): **a** all 104 patients, **b** 32 patients who underwent gastrectomy and **c** 70 patients who underwent gastrojejunostomy

Changes in oral intake after surgery based on GOOSS at each assessment point are shown in *Fig*. 4 *a*. No patient could tolerate solid food at baseline. However, the majority of patients were able to eat solid food 14 days after surgery and this continued thereafter, with 68 patients (65·4 per cent) tolerating a low‐residue or full diet 3 months after surgery. A higher proportion of patients was able to eat solid food at all time points after gastrectomy than after gastrojejunostomy, but this difference was not statistically significant (*Fig*. 4 *b,c*).

### Operative morbidity and mortality

Postoperative complications were identified in 25 patients (24·0 per cent) and the rate of Clavien–Dindo grade III or higher complications was 9·6 per cent (10 patients) (*Table* 2). Reoperation was necessary in two patients (1·9 per cent), in one owing to an abdominal abscess 10 days after gastrojejunostomy and in the other because of duodenal stump leakage 13 days after gastrectomy. There was no recurrent obstruction in need of further intervention during the first admission period. There were six deaths in hospital (5·8 per cent) including two (1·9 per cent) within 30 days of surgery, all considered to reflect disease progression rather than postoperative complications. Median hospital stay for all patients was 13 (i.q.r. 9–18) days.

**Table 2:** Operative morbidity and mortality

	Total	Clavien–Dindo complication grade ≥ III		
		Total	Gastrectomy	Gastrojejunostomy
	(*n* = 104)	(*n* = 104)	(*n* = 32)	(*n* = 70)
Any complication	25	10	4	6
Anastomotic leakage	3	2	2†	0
Abdominal abscess	3	2	1	1†
Pancreatic fistula	2	1	1	0
Wound infection/ dehiscence	6	1	0	1
Pleural effusion	6	1	1	0
Pneumonia	3	0	0	0
Thrombus/embolus	2	1	1	0
Ascites	5	1	0	1
Ileus	5	0	0	0
Gastrointestinal bleeding	5	4	1	3
Reoperation	2		1‡	1§
Death in hospital	6		1¶	5#
30‐day postoperative mortality	2		0	2
Duration of hospital stay (days)*	13 (9–18)		13 (9.5–15.5)	12.5 (9–24)

* Values are median (i.q.r.).

† Grade IIIb.

‡ Duodenal stump leakage on postoperative day (POD) 13;

§ abdominal abscess on POD 10. Disease progression on

¶ POD 137 and

# POD 28, 30, 40, 60 and 67.

## Discussion

Gastric outlet obstruction secondary to incurable primary gastric cancer occurs in almost 20 per cent of patients in the late stages of disease^24^. Although surgical palliation can relieve obstructive symptoms, the decision to offer surgery is influenced by the likely prognosis, and the combined experiences, expectations and preferences of the patient and physician. Factors that determine the ideal treatment for an individual patient are not well defined; prospective studies examining QoL in this patient population are few, with most palliative surgical literature focusing on oesophageal, colorectal, pancreatic and biliary cancers^25^.

Traditional surgical outcome measures, such as morbidity, mortality and survival, do not always sufficiently capture the balance between risk and benefit in palliative surgery. An accurate assessment of health‐related QoL using validated QoL measures is essential to inform clinical decision‐making. Unfortunately, there are few prospective studies using established QoL instruments to assess the impact of surgical palliation in patients with incurable advanced gastric cancer^10^, even though patient‐reported outcome measures are becoming increasingly important in an effort to give patients a better understanding of the treatment and care options available and outcomes, thereby facilitating decision‐making regarding care^26^.

This prospective study provides two quantitative outcome measures of QoL (EQ‐5D™ and QLQ‐STO22) and two observational outcome measures (postoperative improvement in oral intake and safety of surgical intervention) that surgeons and patients can easily understand and incorporate into their decision‐making process. This study did not aim to compare palliative gastrectomy with surgical bypass; the authors were interested primarily in QoL alterations and improvements in oral intake after either gastrectomy or gastrojejunostomy.

Palliative options for gastric outlet obstruction include surgical bypass, endoscopic stenting, percutaneous endoscopic gastrostomy (PEG) or percutaneous endoscopic jejunostomy (PEJ). Patients with advanced disease and a short life expectancy are often managed with medical therapy, endoscopic stenting, PEG or PEJ. A systematic review^27^ and an RCT^28^ comparing enteral stenting *versus* gastrojejunostomy for the palliation of gastric outlet obstruction suggested that stent placement may be associated with more favourable results in patients with a short life expectancy, whereas gastrojejunostomy was preferable in patients with a better prognosis^2^. In the present study, as 88 patients (84·6 per cent) had a PS score of 0–1 and were thus expected to have a relatively longer life expectancy, surgical palliation rather than stent insertion was defined as the protocol treatment.

Patient compliance with follow‐up QoL surveys was excellent for the first month after surgery. After 3 months compliance dropped to around 80 per cent owing to patient death or inability to complete follow‐up questionnaires near the end of life. Other investigators have reported similar difficulties. A randomized trial^29^ examining QoL in patients with unresectable periampullary tumours with or without prophylactic gastrojejunostomy reported a compliance rate of 90 per cent in the first 4 months of follow‐up, but it decreased to 75 per cent in the final 2 months of the study. Likewise, another randomized trial^30^ comparing stenting with surgical bypass for malignant gastric outlet obstruction reported a compliance rate of 57 per cent with follow‐up surveys at 1 month. Loss to follow‐up, owing to disease progression and the burden of questionnaire completion^26^, makes use of patient‐reported outcome measures challenging after palliative surgery, and loss of information from patients near the end life probably removes those with the worst QoL from analysis.

Within this constraint, the present study, nevertheless, provided important insights into the overall well‐being of patients with gastric outlet obstruction after surgical palliation. QoL was maintained at the baseline level for at least 3 months after surgery. Similar QoL after gastrectomy and gastrojejunostomy may help surgeons select the optimal procedures at their discretion. Taking into account that six patients returned no data and 16 had died by the 3‐month follow‐up, the EQ‐5D™ index scores could in reality have deteriorated by 3 months. The proportion of patients with improved or stable EQ‐5D™ scores was therefore compared with the proportion with deteriorated scores or who had died or had no data available at each time point. Surgeons should discuss expectations about QoL impairment with their patients before surgery.

With either intervention, the effect of surgical palliation on gastric‐specific symptoms was more pronounced for dysphagia, reflux, eating restrictions and pain than for anxiety, dry mouth and taste. Both interventions offered almost identical outcomes in all STO22 domains. In the early postoperative period, the majority of patients were able to eat solid food and continued to tolerate this for up to 3 months after surgery, consistent with a previous report^31^.

Studies measuring patient QoL often prefer disease‐specific instruments to generic instruments because the former focus on particular health problems and tend to be more sensitive to clinically important differences. QLQ‐STO22 is a gastric‐specific symptom module generally for use in conjunction with QLQ‐C30 to gain a global picture of QoL. However, using both of these measures requires patients to complete 52 questions, thereby increasing the burden of measuring QoL. This study, therefore, used EQ‐5D™ as the generic instrument rather than QLQ‐C30, although it is acknowledged that EQ‐5D™, because of its relative simplicity, does not address as many domains as QLQ‐C30.

Potential benefits of surgical palliation can be reduced by postoperative complications, and palliative procedures should have low morbidity and mortality rates. Although 5·8 per cent of the patients (6 of 104) died in hospital from disease progression during the primary admission, none died from postoperative complications. The 30‐day postoperative mortality rate of 1·9 per cent was acceptable in comparison with the rate of 0·8 per cent associated with elective potentially curative surgical oncology procedures for advanced gastric cancer in Japan^32^. Given the low rates of postoperative morbidity and mortality in the present study, the extent to which QoL was impaired by the operation is likely to have been small.

The growth of endoscopic procedures has broadened the options for symptom control. Endoscopic stent placement is associated with a shorter hospital stay, trend towards a lower periprocedural mortality rate, early resumption of oral intake and lower costs in patients with malignant gastric outlet obstruction compared with surgical treatment^28^ ^30^, ^33^, ^34^, ^35^, ^36^, ^37^, at the expense of a greater need for reintervention owing to stent occlusion by food impaction, stent migration or tumour ingrowth^27^ ^33^, ^38^.

This study had several limitations. Death and disease‐related difficulties with questionnaire completion affected compliance with follow‐up QoL surveys, although this limitation is inherent to all studies of QoL, but particularly with a rapidly progressing disease such as incurable advanced gastric cancer. QoL follow‐up was limited to just 3 months after surgical palliation, because of the problem of patient loss by this time. The study had intrinsic difficulties in patient accrual in view of its strict eligibility criteria, patient preference and biases of individual clinicians, which may have led to the low annual inclusion rate at each centre. A screening log, including the total number of patients receiving palliative care with reasons for exclusion and other preferred treatment options, was not available.

Surgical palliation maintained patient QoL while improving solid food intake with acceptable surgical toxicity for at least the first 3 months after surgery. The results of this multicentre study are likely to be generalizable to patients undergoing surgical palliation for malignant outlet obstruction for gastric cancer, and should facilitate decision‐making for physicians, patients and carers.
